# ADAR1p110 promotes *Enterovirus* D68 replication through its deaminase domain and inhibition of PKR pathway

**DOI:** 10.1186/s12985-022-01952-6

**Published:** 2022-12-22

**Authors:** Kehan Zhang, Siyuan Wang, Tingting Chen, Zeng Tu, Xia Huang, Guangchao Zang, Chun Wu, Xinyue Fan, Jia Liu, Yunbo Tian, Yong Cheng, Nan Lu, Guangyuan Zhang

**Affiliations:** 1 Pathogen Biology and Immunology Laboratory and Laboratory of Tissue and Cell Biology, Experimental Teaching and Management Center, Chongqing Medical University, Chongqing, China; 2 Department of Pathogen Biology, Basic Medical School, Chongqing Medical University, Chongqing, China; 3 Department of the First Clinical Medicine, Chongqing Medical University, Chongqing, China; 4 Quality Management Section, Chongqing Blood Center, Chongqing, China; 5 Chongqing Better Biotechnology LLC, Chongqing, China; 6 Monitoring On Terrestrial Wildlife-Borne Infectious Diseases, Jinggangshan National Nature Reserve of Jiangxi Province, Ji’an, Jiangxi China

**Keywords:** EV-D68, ADAR1p110, Deaminase domain, dsRBDs, PKR

## Abstract

**Background:**

Severe respiratory and neurological diseases caused by human enterovirus D68 (EV-D68) pose a serious threat to public health, and there are currently no effective drugs and vaccines. Adenosine deaminase acting on RNA1 (ADAR1) has diverse biological functions in various viral infections, but its role in EV-D68 infections remains undetermined.

**Methods:**

Rhabdomyosarcoma (RD) and human embryonic kidney 293 T (293 T) cells, and HeLa cells were used to evaluate the expression level of ADAR1 upon EV-D68 (Fermon strain) and human parainfluenza virus type 3 (HPIV3; NIH47885) infection, respectively. Knockdown through silencing RNA (siRNA) and overexpression of either ADAR1p110 or ADAR1p150 in cells were used to determine the function of the two proteins after viral infection. ADAR1p110 double-stranded RNA binding domains (dsRBDs) deletion mutation was generated using a seamless clone kit. The expression of ADAR1, EV-D68 VP1, and HPIV3 hemagglutinin–neuraminidase (HN) proteins was identified using western blotting. The median tissue culture infectious dose (TCID_50_) was applied to detect viral titers. The transcription level of EV-D68 mRNA was analyzed using reverse transcription-quantitative PCR (RT-qPCR) and the viral 5′-untranslated region (5′-UTR)-mediated translation was analyzed using a dual luciferase reporter system.

**Conclusion:**

We found that the transcription and expression of ADAR1 was inhibited upon EV-D68 infection. RNA interference of endogenous ADAR1 decreased VP1 protein expression and viral titers, while overexpression of ADAR1p110, but not ADAR1p150, facilitated viral replication. Immunofluorescence assays showed that ADAR1p110 migrated from the nucleus to the cytoplasm after EV-D68 infection. Further, ADAR1p110 lost its pro-viral ability after mutations of the active sites in the deaminase domain, and 5′-UTR sequencing of the viral genome revealed that ADAR1p110 likely plays a role in EV-D68 RNA editing. In addition, after ADAR1 knockdown, the levels of both phosphorylated double-stranded RNA dependent protein kinase (p-PKR) and phosphorylated eukaryotic initiation factor 2α (p-eIF2α) increased. Attenuated translation activity of the viral genome 5′-UTR was also observed in the dual-luciferase reporter assay. Lastly, the deletion of ADAR1p110 dsRBDs increased the level of p-PKR, which correlated with a decreased VP1 expression, indicating that the promotion of EV-D68 replication by ADAR1p110 is also related to the inhibition of PKR activation by its dsRBDs. Our study illustrates that ADAR1p110 is a novel pro-viral factor of EV-D68 replication and provides a theoretical basis for EV-D68 antiviral research.

## Introduction

Enterovirus (EV)-D68 has been a public health concern since an outbreak of respiratory illness in the United States in 2014 [1]. Similar to other picornaviruses, EV-D68 is a single-stranded, positive-sense RNA virus, with a genome of approximately 7500 nts, consisting of a 5′-untranslated region (UTR) that contains an internal ribosome entry site (IRES), an open reading frame (ORF), and a 3′-UTR that contains a poly A tail [2]. The EV-D68 genome encodes a variety of structural (VP1, VP2, VP3, and VP4) and non-structural (2A, 2B, 2C, 3A, 3B, 3C, and 3D) proteins [3]. Unlike other enteroviruses, EV-D68 mainly causes respiratory tract infections [4], especially lower respiratory tract infections in infants and young children. In addition, adults with hematological malignancies or those who have received hematopoietic cell transplants can be infected with EV-D68 [5]. Infection with EV-D68 are correlated with acute flaccid myelitis (AFM), which may cause limb pain and autonomic dysfunction, and even paralysis in severe cases [6]. However, currently, there are no vaccines or drugs against EV-D68. Therefore, exploring the interaction between EV-D68 infection and relative host factors is of great clinical significance for the prevention and treatment of EV-D68 infection, and, furthermore, to reinforce the protection of public health, especially of infants and young children.

A variety of double-stranded RNA-binding proteins with important cellular functions have been identified [7]. Among them, the adenosine deaminase acting on RNA (ADAR) protein family can bind to specific regions of double-stranded RNA and catalyze the generation of inosine residues from adenosine residues, leading to A-to-I RNA editing [7, 8]. In addition, ADAR proteins are associated with immune system regulation, mRNA splicing, microRNA processing, and RNA silencing [9–11]. In mammalian cells, the ADAR protein family consists of three members, ADAR1, ADAR2, and ADAR3, all of which contain a catalytic deaminase domain, which is responsible for RNA editing activity, and double-stranded RNA binding domains (dsRBDs) [12]. ADAR1 is the most commonly studied ADAR protein in viral infections and can be divided into two subtypes, ADAR1p150 and ADAR1p110. ADAR1p150 is an interferon (IFN)-induced protein that is distributed in the nucleus and cytoplasm, while ADAR1p110 is constitutively expressed in the nucleus.

ADAR1 was initially identified as an antiviral factor that is mainly related to RNA editing activity. It has been reported that ADAR1-mediated A/I hypermutation in persistent measles virus(MeV) infection attenuates virus-induced cytopathic effects [13]. A-to-I editing by ADAR1 greatly antagonizes the biogenesis of encephalomyocarditis virus circular RNAs [14]. However, increasing evidence suggests that ADAR1 can also act as a pro-viral factor. Reportedly, ADAR1 can directly interact with influenza A NS1 and NS2 or dengue virus NS3 proteins to promote viral replication, and the editing activity of ADAR1 is also enhanced by these viral proteins [15]. In a study on hepatitis B virus (HBV), ADAR1 lost its pro-viral ability after mutation of the active site in its deaminase domain, suggesting that ADAR1 promotes HBV replication through its deaminase domain [16]. In Zika virus (ZIKV) and human immunodeficiency virus type I (HIV-1), ADAR1 can inhibit the activation of protein kinase R (PKR), thereby inhibiting the phosphorylation of eIF2α and promoting viral protein synthesis [17, 18]. Recent research has demonstrated that ADAR1 exhibits double-edged effects during the early or middle-late stages of Coxsackie virus B3 infection [19].

Although the function of ADAR1 in various viral infections has been established, its role in EV-D68 infection remains unknown. Our findings show that EV-D68 infection can suppress ADAR1 expression. Moreover, ADAR1p110, but not ADAR1p150, promoted EV-D68 replication. Further analysis revealed that the pro-viral effect of ADAR1p110 was related to both its enzymatic activity and inhibition of the PKR pathway by dsRBDs. In summary, this study clarifies the mechanism by which ADAR1 promotes EV-D68 replication, providing a new target and theoretical basis for the prevention and treatment of EV-D68 infection.

## Materials and methods

### Cells, viruses and reagents

RD cells, 293 T cells, HeLa cells and ADAR1 stable knockdown cell line (ADAR1 KD) were cultured in Dulbecco’s modified Eagle’s medium (DMEM, HyClone, USA) with 10% fetal bovine serum (FBS, Gibco, USA) and 1% penicillin and streptomycin (HyClone, USA) at 37 °C with 5% CO_2_. EV-D68 (Fermon strain) was kept in our lab and propagated in RD cells. HPIV3 (NIH47885) was kindly granted by Professor Mingzhou Chen of Wuhan University and propagated in HeLa cells by inoculation at a multiplicity of infection (MOI) of 0.1.

### Plasmids mediated gene expression and siRNA mediated gene targeting

Plasmids pCAGGS, pCAGGS-DDK-ADAR1p150 (termed as ADAR1p150 or DDK-ADAR1p150), plasmids M07, M07-HA-ADAR1p110 (termed as ADAR1p110 or HA-ADA1p110), M07-HA-ADAR1p110-H910A, M07-HA-ADAR1p110-C996A and M07-HA-ADAR1p110-C1036A (termed as p110-H910A, p110-C996A and p110-C1036A) were described previously [20]. And pCAGGS plasmids were used as control. The plasmids used for expressing dsRBDs deleted ADAR1p110 (termed as p110-△RBD) were generated by amplifying plasmid M07-HA-ADAR1p110 with the primers ADAR1-Drbd-F: ATTGGGGAGAACGAGAAGGCA and ADAR1-Drbd-R: CTTCTCGTTCTCCCCAATGTTCTTCAGCTGGCACTCTG, then ligated the PCR product by using seamless ligase kit (Biorun, China) and transformed to the DH5α competent cells. To generate the dual-luciferase reporter plasmids (termed as pSiCheck2-5′UTR), the 5′-UTR, including the first 19 amino acid coding regions in the ORF of EV-D68 (Fermon strain), was amplified using a primers pair, 5UTR-F: AAAGCTCTTCATAGTTAAAACAGCTCTGGGGTTG and FermVP4-R: AAAGCTCTTCAGGCGGTGGCTAGCGCAATGTTAGCATTCTCA. pSiCheck2 plasmids were amplified using the primers, Fluc-F: AAAGCTCTTCAGCCGATGCTAAGAACATTAAG and Rluc-R: AAAGCTCTTCACTAGAATTACTGCTCGTTCTTCAGCA. The two fragments were ligated based on the protocols of Golden Gate clone shuffling method [21, 22]. Small interfering RNA targeting ADAR1 (si-ADAR1) was purchased from Guangzhou RiboBio (RiboBio, China). All the plasmids and siRNAs were transfected into cells using Lipofectamine 3000 reagent (Invitrogen, USA) following the manufacturer’s instructions.

### Viral infection and viral titers measurement

RD cells were seeded in 6-well plates. When the cell density reached 40–50%, EV-D68 inoculum was added and incubated at 37 °C with 5% CO_2_. After 1.5 h, the supernatant was replaced by DMEM containing 4% FBS. According to different experimental requirements, cell samples were collected after indicated hours. For HPIV3 infection, the same operations were performed on HeLa cells. The titer of virus was determined by TCID_50_ method. Specifically, RD cells were cultured in 96-well plates. When cell density reached 30–40%, the original medium was discarded and the cells were washed by PBS for two times. Meanwhile, the virus stock for testing was diluted in a tenfold gradient in DMEM medium, from 10^–3^ to 10^–8^. The PBS medium in the wells of 96 plates was discarded and then the diluted viral inoculums were added into the wells in order. Each dilution was replicated for 3 wells. Two hours later, the supernatant was replaced with DMEM medium contains 4% FBS. The cells were observed for 3–5 consecutive days to record the cytopathic effect (CPE). TCID_50_ was calculated by Spearman-Karber method. The formula is lgTCID_50_ = L + D (S-0.5). L is the logarithm of the lowest dilution, D is the dilution factor and S is the ratio of positive wells combined.

### Western blotting

The infected or transfected cells were washed and harvested with pre-chilled phosphate buffered saline (PBS), then centrifuged at 13,000 rpm for 1 min, thereafter whirled with TNE buffer (50 mM Tris–Cl [pH 7.4], 150 mM NaCl, 2 mM EDTA [pH 8.0], 0.1% 2-mercaptoethanol and protease inhibitor cocktail) to lyse cell debris for 30 min. Cell lysates were centrifuged at 13,000 rpm for 30 min at 4 °C. The protein-containing supernatant was mixed with 5 × SDS-PAGE loading buffer, boiled at 100 °C for 10 min, then subjected to a 10% sodium dodecyl sulfate–polyacrylamide gel and electro-blotted the samples onto a PVDF membrane. Skim milk was dissolved with PBS with Tween 20 (1/1000 Tween 20) and the membrane was blocked for 30 min at room temperature. After that, the PVDF membrane was incubated with primary antibody for 1.5 h and secondary antibody for 45 min. The primary antibodies used were donkey anti-HPIV3 (1:2500, Abcam, United Kingdom), rabbit anti-ADAR1 (1:500, Santa Cruz, USA), rabbit anti-HA(1:4000, Proteintech, China), rabbit anti-DDK(1:4000, Proteintech, China), rabbit anti-beta-actin (1:1000, Proteintech, China), mouse anti-PKR (1:4000,huabio, China), mouse anti-P-PKR (1:2000,huabio,China), mouse anti-eIF2α(1:2000, huabio, China) and mouse anti-P-eIF2α(1:2000,huabio, China). HRP-conjugated goat anti-mouse IgG (1: 5000, Sigma, USA), HRP-conjugated goat anti-rabbit IgG (1:5000, Sigma, USA) and HRP-conjugated goat anti-donkey IgG (1:5000, Sangon, China) were used as secondary antibodies.

### RT-qPCR assay

RD cells were digested with TRIzol reagent (Beyotime, China) and total cellular RNA was extracted. Total RNA was reverse transcribed into cDNA using PrimeScript RT reagent kit with gDNA Eraser (TaKaRa, Japan). The amount of ADAR1 and beta-actin were quantified by using BioRun ChemoHS qPCR Mix (SYBR) (Biorun, China) and Light Cycler (Roche, Switzerland). Data shown are the relative abundance of ADAR1 RNA with normalization to the housekeeping gene beta-actin. The primers used for RT-qPCR were as follows: Q-actin-F: TGAAGTGTGACGTGGACATCCG, Q-actin-R: GCTGTCACCTTCACCGTTCCAG and primers set for ADAR1 were described before [20].

### Immunofluorescence microscopy

HeLa cells were grown to 30–40% confluency in 12-well plates plated with cell slides and transfected of ADAR1 expression plasmids or infected with EV-D68 as described above. 24 h later, slides were taken out and washed three times with 4 °C PBS, fixed with 4% paraformaldehyde for 20 min, permeabilized with 0.2% Triton X-100 for 25 min, and blocked with 3% bovine serum albumin (BSA) for 30 min. Primary antibody was incubated for 1.5 h at room temperature. The primary antibody were rabbit anti-HA tag (1:100, Proteintech, China), rabbit anti-DDK tag (1:50, Proteintech, China) and mouse J2 anti-dsRNA (1:200, Scicons, Netherlands). Cells were subsequently washed 3 times with 1% BSA and incubated with secondary antibody for 45 min at room temperature. The secondary antibody used were Alexa Fluor 488 conjugated goat anti-rabbit IgG and Alexa Fluor 568 conjugated goat anti-mouse IgG (1:1000, Thermo, USA). Nuclei were stained with DAPI (SolarBio, China). Cells were observed by immunofluorescence microscopy (Nikon Ts2-FL, Japan).

### Luciferase assay

The luciferase activity assay was performed according to the kit instructions (Promega, USA). Briefly, 24 h after transfection of pSiCheck2-5’UTR with firefly and renilla reporter gene luciferase expression plasmid, medium was removed and 400 μL of 1 × Lysis Reagent was dispensed into each cell. Pellet the debris by centrifugation at 12,000 rpm for 1 min and transfer the supernatant to a new tube. Mix 20 μL of cell lysates with 100μL of luciferase detection reagent and measure the resulting light intensity using the SpectraMax-iD5 (Molecular Devices, USA).

### Sequencing of EV-D68 5’-UTR region

After infection with EV-D68, cellular total RNA was extracted with Trizol reagent (Beyotime, China) and reverse transcribed was performed by using reverse transcription kit (Takara, Japan). Thereafter, the EV-D68 5’-UTR region was amplified by using the 2 × PFU MasterMix (TIANGEN, China). A primer set complementary to the corresponding region was as follows: 5UTR-SF: TAAAACAGCTCTGGGGTTG, 5UTR-SR: GCATTCTCATGAGTTCCAG. At last, PCR products were analyzed by Sanger sequencing.

### Statistical analysis

The amounts of protein in cell lysates were estimated based on the density of protein bands using Bio-Rad software Quantity One-4.6.2. The amount of ADAR1/beta-actin, VP1/beta-actin, HN/beta-actin in the corresponding lysates are normalized to the value obtained in the control group which is set to 1.0. The phosphorylation level of either PKR or eIF2a were quantified as the amount of p-PKR/PKR or p-eIF2a/eIF2a. The results are shown as average with standard error of mean (SEM), and GraphPad Prism 9.0 was used to analyze the statistics. Statistical analysis was performed using Student’s *t*-test, and *p* values less than 0.05 were considered significantly different and indicated by asterisks in the figures.

## Results

### EV-D68 infection inhibits the expression of ADAR1

**Fig. 1:**
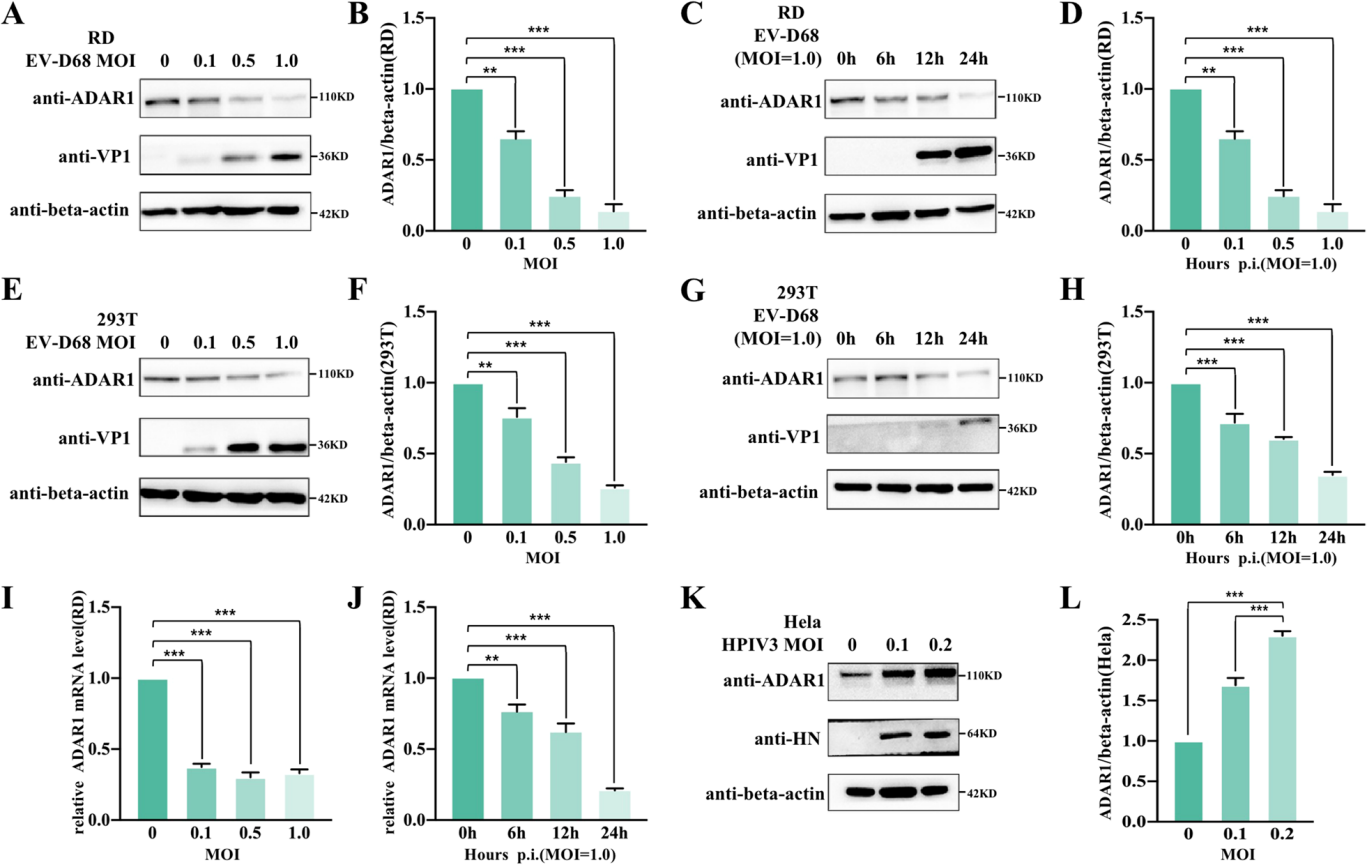
EV-D68 infection inhibits the expression of ADAR1. (**A**) RD cells were infected with EV-D68 at MOI of 0, 0.1, 0.5 and 1.0 for 24 h, and the cells were lysed for western blotting to detect the expression level of viral protein VP1 and endogenous protein ADAR1. Beta-actin was probed as the internal control. (**B**) ADAR1/beta-actin for each groups were calculated as described in “Materials and Methods” section. (**C**, **D**) RD cells were infected with EV-D68 at MOI of 1.0 for 0, 6, 12 and 24 h. Then the expression of viral VP1 and endogenous ADAR1 in cell lysates were detected, and ADAR1/beta-actin for each groups were calculated as described above. 293 T cells were infected with EV-D68 either at MOI of 0, 0.1, 0.5 and 1.0 for 24 h (**E**, **F**) or at MOI of 1.0 for 0, 6, 12 and 24 h (**G**, **H**). VP1 and endogenous ADAR1 were detected, and ADAR1/beta-actin were quantified as described above. (**I**) ADAR1 mRNA levels in RD cells from (**A**) were detected via RT-qPCR as described in “Materials and Methods”section. Cellular beta-actin mRNA was used as control. (**J**) ADAR1 mRNA levels in RD cells from (**C**) was detected by RT-qPCR as described above. (**K**, **L**) Hela cells were infected with HPIV3 at MOI of 0, 0.1 and 0.2 for 36 h, and the samples were collected, viral HN, endogenous ADAR1 and beta-actin were detected by western blotting. ADAR1/beta-actin for each groups were calculated as described above. In all the panels for western blotting, the relative molecular mass was marked besides each band. All data above were shown as mean ± SEM of n = 3 replicates, student’s t-test: **p < 0.01; ***p < 0.001

To verify the role of ADAR1 in EV-D68 infection, we measured the endogenous ADAR1 expression levels after viral infection in either RD or 293 T cells. Our result in RD cells showed a gradual decrease in protein expression of ADAR1 with an increase in MOI. At a MOI of 1.0, the protein expression of ADAR1 was only 14% of the protein expression in uninfected control cells (Fig. 1A, B). Moreover, ADAR1 expression was considerably reduced to 13% compared to that in uninfected cells at 24 h post-infection (Fig. 1C, D). Only the band of endogenous ADAR1p110 was detected at 110 KD, while that of ADAR1p150 was too weak to be detected. Similarly, in 293 T cells, the reduction of ADAR1 level was dose- (Fig. 1E, F) and time-dependent (Fig. 1G, H) with viral infection. Moreover, RT-qPCR results showed that the mRNA level of ADAR1 decreased after viral infection (Fig. 1I, J), indicating that ADAR1 is probably regulated at the transcriptional level after viral infection. Contrary to the results for EV-D68, after infection with HPIV3, another respiratory virus, the protein expression levels of ADAR1 showed an ascending trend (Fig. 1K, L). The above results suggest that ADAR1 could be regulated after EV-D68 or HPIV3 infection, but the role of ADAR1 in viral infection is unknown.

### ADAR1p110, but not ADAR1p150, promotes EV-D68 replication

**Fig. 2:**
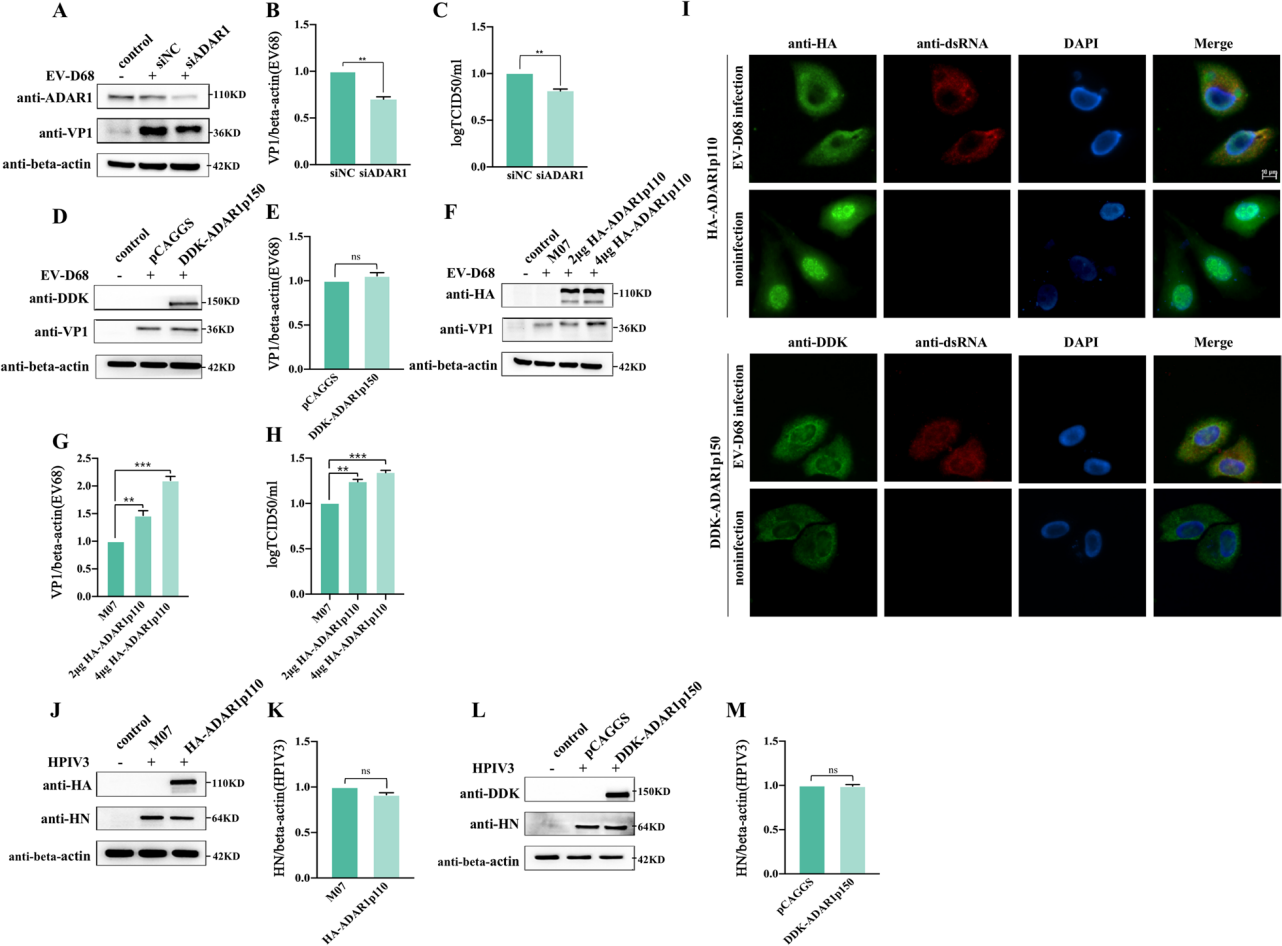
ADAR1p110 but not ADAR1p150 promotes EV-D68 replication. (**A**) 293 T cells were transfected with either siADAR1 or siNC, respectively. 48 h later, the transfected cells were infected with EV-D68 at MOI of 0.2. At 24 h after infection, cells were lysed and the expression levels of ADAR1 and viral protein VP1 were determined by western blotting, the supernatant was collected for viral titer assay. (**B**) The amount of VP1/beta-actin in (**A**) were quantified as described above. (C) Viral titers of the cell supernatant from (**A**) were determined by TCID_50_. (**D**) 293 T cells were transfected with plasmids expressing DDK-ADAR1p150 (4ug) or vector pCAGGS, 24 h later, the transfected cells were infected with EV-D68 at MOI of 0.2. The cells were collected and the expression of VP1, DDK-ADAR1p150 and beta-actin were determined by western blotting after 24 h infection. (**E**) VP1/beta-actin of each group were quantified as described above. (**F**) 293 T cells were transfected with 2 or 4 μg of plasmids expressing HA-ADAR1p110 or empty vector M07, then the cells were infected with EV-D68 as above. HA-ADAR1p110 and VP1 were determined by western blotting. (**G**)VP1/beta-actin for groups in (**F**) were quantified as above. (**H**) The cell supernatant from (**F**) was collected and viral titers were determined by TCID_50_. (**I**) Either HA-ADAR1p110 or DDK-ADAR1p150 expression plasmids were transfected into Hela cells, separately. After 24 h post transfection, cells were infected with EV-D68 (MOI 1.0). At 24 h after infection, cells were fixed and HA-ADAR1p110 was stained with rabbit HA primary antibody and goat anti-rabbit AF488, DDK-ADAR1p150 was stained with rabbit DDK primary antibody and goat anti-rabbit AF488, viral RNAs were stained with mouse J2 antibody and goat anti-mouse AF568. Nuclei were counterstained with DAPI. Scale bar is 10 μm. (**J, K**) Hela cells were transfected with plasmids expressing HA-ADAR1p110. 48 h later, cells were infected with HPIV3 at MOI of 0.1 for 24 h. The HA-ADAR1 and viral protein HN levels were determined by western blotting and HN/beta-actin were quantified as above. (**L, M**) Hela cells were transfected with plasmids expressing DDK-ADAR1p150 and infected with HPIV3 as above, DDK-ADAR1p150 and HN in cell lysates were detected and HN/beta-actin were quantified as above. In all the panels for western blotting, the relative molecular mass was marked besides each band. All data above were shown as mean ± SEM of n = 3 replicates, student’s t-test: **p < 0.01; ***p < 0.001; NS, not significant

To assess the effect of ADAR1 on EV-D68 infection, endogenous ADAR1 was knocked down using siRNA. The results showed that, compared to the siNC group, VP1 protein expression was significantly reduced to 73% (Fig. 2A, B) and the virus yields were also lower (Fig. 2C) in the siADAR1 group, suggesting that ADAR1 could facilitate EV-D68 replication. To identify the isoforms of ADAR1 that exert pro-viral effects, we overexpressed DDK-ADAR1p150 and HA-ADAR1p110 in 293 T cells. Compared with the pCAGGS control plasmid, no obvious changes in EV-D68 VP1 protein expression were observed after the overexpression of DDK-ADAR1p150 (Fig. 2D, E). Conversely, the expression of VP1 gradually increased with the overexpression of HA-ADAR1p110 (Fig. 2F, G), and upon transfection with 2 and 4 μg of HA-ADAR1p110-expressing plasmids, VP1 expression increased by 11 and 27% respectively. The virus titers also increased, correspondingly (Fig. 2H). Previous studies have shown that ADAR1p110 is constitutively expressed and mainly localized in the nucleus [23, 24]. Our immunofluorescence assay results showed that HA-ADAR1p110 aggregated in the nuclei of uninfected cells in the form of dots. However, upon EV-D68 infection, the punctuation in the nucleus disappeared and diffused into the cytoplasm, while the addition of EV-D68 did not affect the cytoplasm-dominant localization of DDK-ADAR1p150 (Fig. 2I). As expected, ADAR1p110 could migrate from the nucleus to the cytoplasm during EV-D68 infection, yet ADAR1p150 could not. In contrast, in the case of HPIV3, the expression of viral HN protein remained unaffected after overexpression of either HA-ADAR1p110 or DDK-ADAR1p150 (Fig. 2J–M). In conclusion, these observations demonstrate that ADAR1p150 does not affect the replication of EV-D68 or HPIV3, while ADAR1p110 plays an important role during EV-D68 infection.

### The pro-viral effect of ADAR1p110 on EV-D68 is related to its deaminase domain

**Fig. 3:**
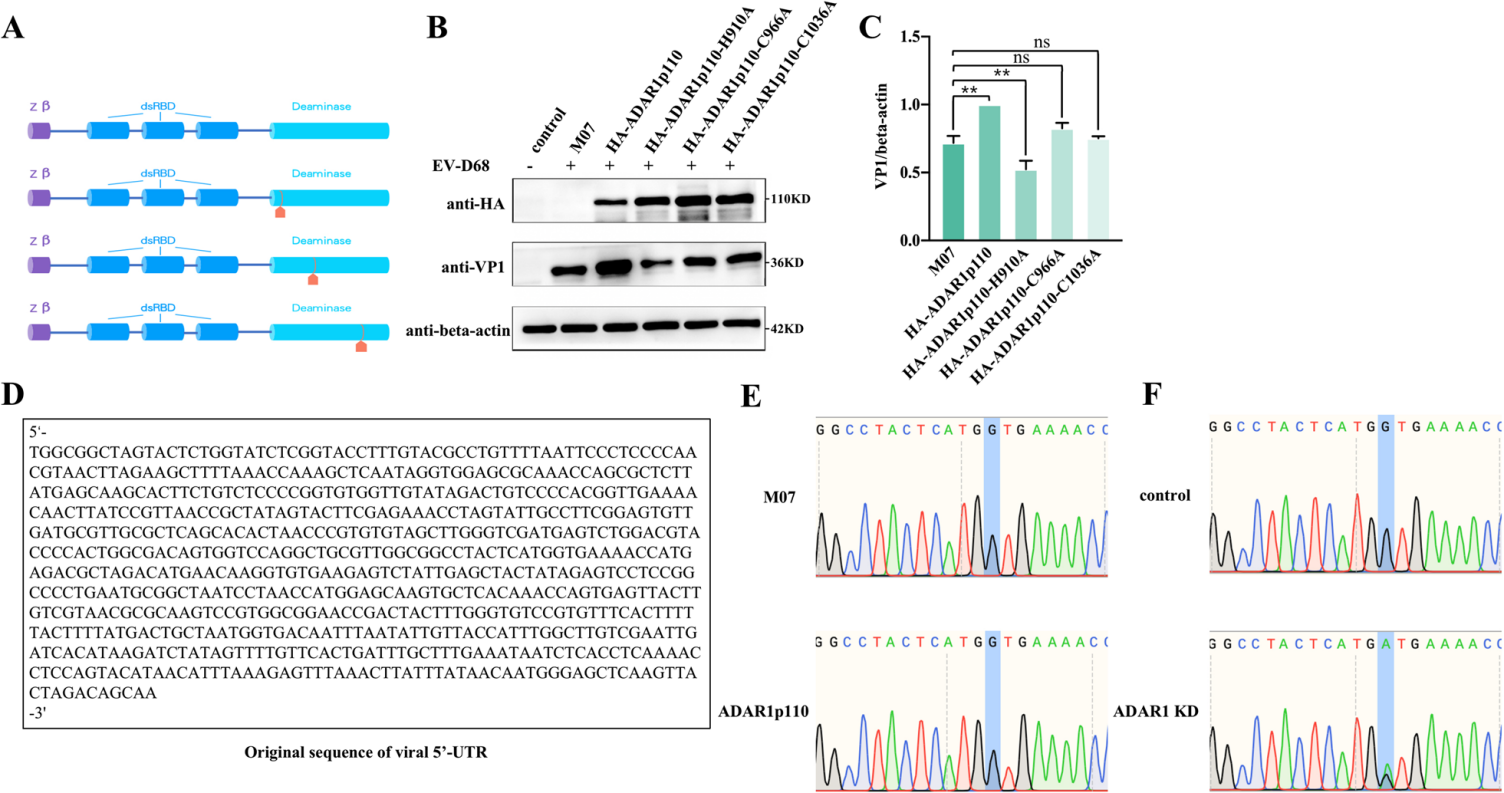
The pro-viral effect of ADAR1p110 on EV-D68 is related to its deaminase domain. (**A**) Schematic structural illustration of ADAR1p110 and its three key amino acid sites for the enzyme activity in the deaminase domain. (**B, C**) 293 T cells were transfected with plasmids M07, M07-HA-ADAR1p110, M07-HA-ADAR1p110-H910A, p110-C996A and p110-C1036A, separately. 48 h later, cells were infected with EV-D68 at MOI of 0.2. After 24 h infection, the expression levels of HA-ADAR1, ADAR1 mutants and VP1 in cell lysates were detected by western blotting and the relative molecular mass was marked besides each band. VP1/beta-actin of each group were quantified as described above. (**D**) The original sequence of viral 5’-UTR was presented in the box. (**E**) 293 T cells were transfected with either plasmid expressing HA-ADAR1p110 or the control plasmid M07. 48 h later, the cells were infected with EV-D68 at MOI of 0.2. The cells were collected at 24 h post-infection, and total cellular RNA was extracted for RT-qPCR. Mutations in viral 5’-UTR were determined by Sanger sequencing. DNA sequencing chromatograms of PCR products are shown. (**F**) After 15 generations of persistent infection in ADAR1 knockdown (ADAR1 KD) 293 T or normal 293 T cells, the 5’-UTR mutation of EV-D68 was determined as described above. DNA sequencing chromatograms of RT-qPCR products are shown. All data above are shown as mean ± SEM of n = 3 replicates, student’s t-test: **p < 0.01;***p < 0.001; NS, not significant

To explore the mechanism by which ADAR1p110 promotes EV-D68 replication, three constructs with point mutations in active sites of the ADAR1p110 deaminase domain were introduced: p110-H910A, p110-C996A, and p110-C1036A (Fig. 3A). All of these mutants had lost their catalytic activity to varying degrees [25]. The results validated the pro-viral effect of wild-type ADAR1p110, while VP1 expression in the p110-C996A and p110-C1036A groups was comparable to that in the control M07 group, and the VP1 level after p110-H910A overexpression was lower than that in the M07 group (Fig. 3B, C). Since p110-C996A and p110-C1036A partially lost the ability to promote EV-D68 replication, and p110-H910A showed an inhibitory effect on viral infection, we may conclude that the deaminase domain of ADAR1p110 plays a key role in EV-D68 infection. To verify whether ADAR1p110 directly edits the viral genome during EV-D68 replication, 293 T cells were transfected with an HA-ADAR1p110 overexpression plasmid and its control M07, followed by infection with EV-D68, and amplification of the 5′-UTR region of the virus and Sanger sequencing. Compared to the original sequence of the viral 5′-UTR (Fig. 3D), no mutations occurred either in the HA-ADAR1p110 transfected group (Fig. 3E, bottom panel) or in the control group (Fig. 3E, top panel). Subsequently, normal 293 T cells and ADAR1 KD cells were infected with EV-D68. After 15 passages of continuous infection, the 5′-UTR of the viral genome was amplified and sequenced as described above. The results showed that, when passaged in the ADAR1 KD cell line, partial G-to-A mutations occurred at position 352 of the viral genome 5′-UTR (Fig. 3E, right, bottom panel). No base mutation was detected in normal 293 T cells (Fig. 3E, top panel). These results indicate that the promotion effect of ADARp110 on EV-D68 is attributed to its deaminase domain and may be related to its RNA editing activity.

### ADAR1p110 promotes EV-D68 replication by inhibiting the activation of PKR pathway

**Fig. 4:**
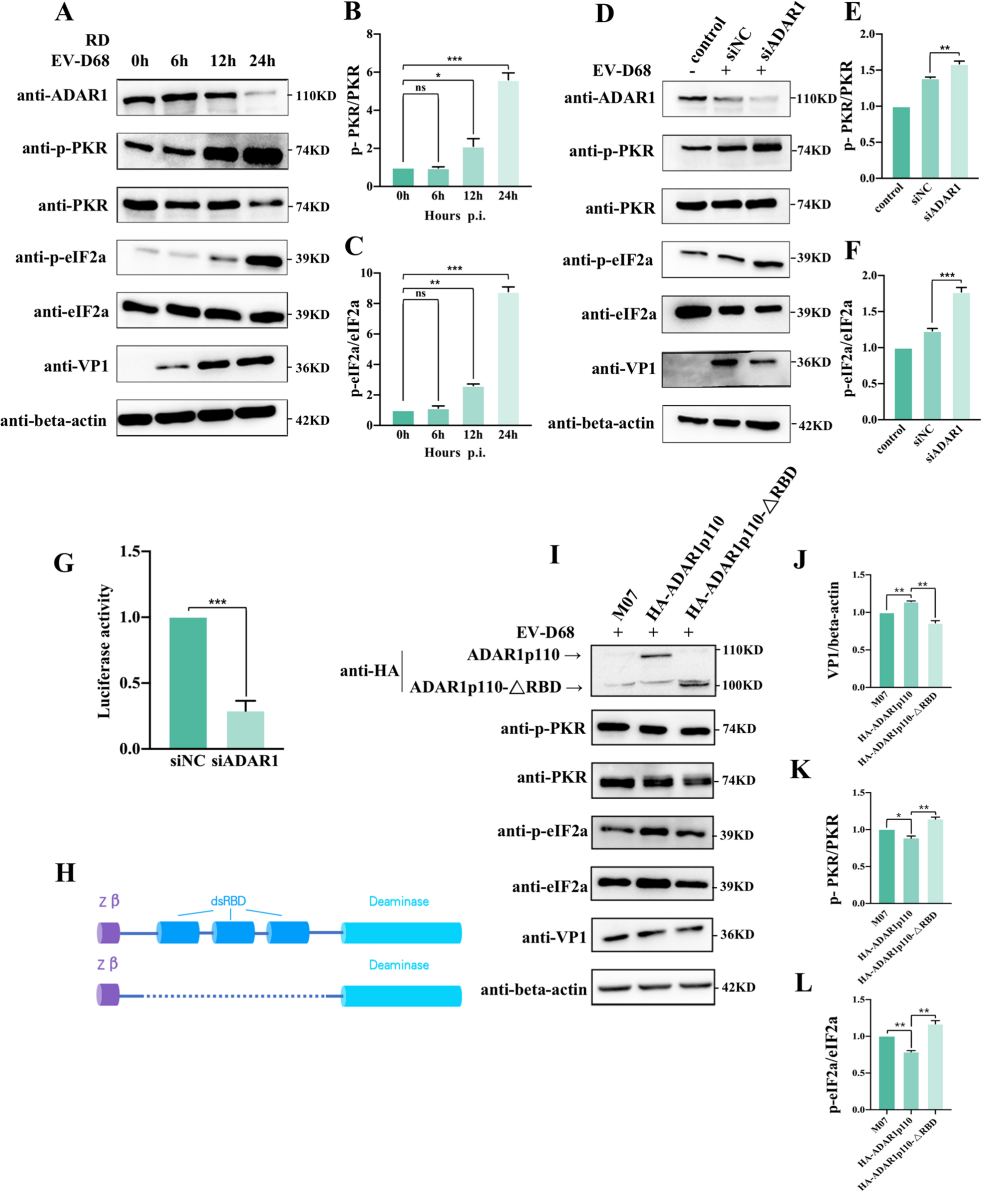
ADAR1p110 promotes the EV-D68 replication by inhibiting the activation of protein kinase PKR. (**A**) Time gradient of EV-D68 infection experiments were performed in 293 T cells as previously described. The expression level of original ADAR1, viral VP1, eIF2a and p-eIF2a, PKR and p-PKR, and the control protein β-actin were detected by western blotting and the relative molecular mass was marked besides each band. (**B**, **C**) The relative phosphorylation levels of PKR and eIF2α were quantified as described before. (D) ADAR1 were knocked down in 293 T cells by transfection of siADAR1 or siNC as described before, and then infected with EV-D68 (MOI = 1.0) for 24 h. The expression level of endogenous ADAR1, viral VP1, eIF2a and p-eIF2a, PKR and p-PKR, and the control β-actin were detected by western blotting. (**E**, **F**) The phosphorylation levels of PKR and eIF2α were quantified as described before. (**G**) The dual luciferase plasmid which containing the firefly luciferase gene under the control of EV-D68 5’-UTR region and the renilla luciferase gene was co-transfected with siADAR1 or siNC. 24 h later, dual luciferase assays were performed as described before. (**H**) Schematic illustration of wild type ADAR1p110 (upper) and the RBD deleted ADAR1, named ADAR1p110-△RBD (lower). (**I**) Plasmids expressing wild type ADAR1p110, ADAR1p110-△RBD, or empty vector M07 was transfected into 293 T cells. 48 h later, cells were infected with EV-D68 as described in “Materials and Methods” section. The expression levels of viral VP1, ADAR1p110, ADAR1p110-△RBD, beta-actin and the phosphorylation level of PKR and eIF2α were determined by western blotting. (**J, K, L**) The expression level of viral protein VP1 and the phosphorylation level of PKR and eIF2α were calculated as described before. In all the panels for western blotting, the relative molecular mass was marked besides each band. All data above were shown as mean ± SEM of n = 3 replicates, student’s t-test: *p < 0.05, **p < 0.01, ***p < 0.001, NS, not significant

ADAR1 has been reported to inhibit PKR phosphorylation during ZIKV and HIV-1 infections, resulting in decreased p-eIF2α levels and increased viral protein synthesis [17, 26]. To test whether ADAR1 promotes EV-D68 replication by regulating the PKR pathway, we measured the phosphorylation level of PKR and eIF2α during viral infection. Western blotting data showed that, with the accumulation of time post-EV-D68 infection, the expression of ADAR1 decreased as before; in contrast, the level of p-PKR gradually increased, and the level of p-PKR/PKR in the 24 h-infection group increased fourfold compared to that in the non-infection group. Consistently, the level of p-eIF2α gradually increased (Fig. 4A, B, and C). Furthermore, after knocking down of endogenous ADAR1, the phosphorylation levels of PKR and eIF2α both increased. In contrast, viral VP1 protein expression was significantly reduced (Fig. 4D, E, and F). To determine whether viral 5′-UTR-mediated translation was regulated by the phosphorylation of PKR and eIF2α, we performed dual luciferase reporter gene assays. The results showed that the expression of the viral 5′-UTR-mediated luciferase reporter gene was significantly reduced after ADAR1 knockdown, suggesting that ADAR1 could regulate the translation of the viral ORF through the PKR pathway and may thus promote viral infection (Fig. 4G). Given that the dsRBDs of ADAR1 can directly interact with PKR [26, 27], it is plausible that ADAR1p110 regulates PKR activation via its dsRBDs during EV-D68 infection. To test this hypothesis, ADAR1p110-△RBDs were expressed in 293 T cells (Fig. 4H). The transfected cells were then infected with EV-D68. The results showed that, compared to the wild-type ADAR1p110, overexpression of p110-△RBD led to significantly increased phosphorylation levels of PKR and eIF2α but impaired viral VP1 expression (Fig. 4I–L); p-PKR/PKR increased by 30% and p-eIF2α/eIF2α by 20%. Thus, it can be concluded that ADAR1p110 inhibits the phosphorylation of PKR through its dsRBDs, thereby inhibiting the phosphorylation of eIF2α and, ultimately, facilitating viral protein translation and exerting a pro-viral effect.

## Discussion

ADAR1 can catalyze the conversion of adenosine to inosine on double-stranded RNA, thereby regulating endogenous and exogenous RNA functions in cells, and plays a key role in development, cancer occurrence, and antiviral responses [28]. Previous reports have revealed that ADAR1 assumes diverse roles in viral infection depending on virus type [17, 29, 30]. The effect of ADAR1 on *Enterovirus* infection has only been reported for CV-B3 [19]. Here, we report that the promotion of EV-D68 replication by ADAR1 correlates with its RNA-editing activity and inhibition of PKR activation.

We found that EV-D68 significantly inhibited the expression of ADAR1; however, the expression of the ADAR1p110 protein increased after HPIV3 infection. In addition, ADAR1p110 can promote the replication of EV-D68 but has no effect on HPIV3 infection, which is consistent with previous reports regarding the diverse role of ADAR1 in different viral infections. However, how do host cells regulate ADAR1 after viral infection? The related mechanisms and pathways remain to be studied. Notably, ADAR1p150 expression was not detected after either EV-D68 or HPIV3 infection. In line with this notion, ADAR1p150 is mainly induced by IFN, while EV-D68 2A and 3C proteases can inhibit the production of type I IFN[31, 32], and the C protein of the Paramyxoviridae family generally bind and interfere with several cytoplasmic proteins which are required for IFN induction [33]. Therefore, we speculate that interruption of the generation of IFN, triggered by EV-D68 and HPIV3 viral proteins, was responsible for the undetected ADARp150. ADAR1 was initially studied as an RNA editing enzyme. For instance, A-to-I editing at the 3′-UTR region of Marburg and Ebola viruses mediated by ADAR1 promoted translation and thus viral replication [34]. The deaminase domain of ADAR1p110 is critical for facilitating HBV replication [20], and ADAR1-mediated HBV RNA editing can help the virus evade recognition by the immune system [35]. Deep sequencing of nasopharyngeal swabs from SARS-CoV-2 patients showed that ADAR1 editing reduced both infectivity and lethality of the virus [36]. The above studies suggest that the RNA editing activity of ADAR1 also plays different roles in different viruses. In this study, the three point-mutants of the ADAR1p110 deaminase domain lost the ability to promote viral replication, and the p110-H910A mutant had an inhibitory effect on viral replication. A similar phenomenon has been observed in HBV, in which the positive effect of ADAR1 on the virus was inhibited after mutation of the ADAR1 deaminase domain [20]. However, the underlying mechanism remains to be elucidated. A previous study on HPIV3 found that A-to-I base substitutions could be detected in the viral genome using PCR amplification and sequencing after 29 months of persistent infection in Lilly Laboratories Cell-Monkey Kidney 2 (LLC-MK2) cells [37]. However, in our findings, A-to-I editing could not be detected in the 5′-UTR of EV-D68 after transient transfection of ADAR1p110, which may be due to the low expression level of transient transfection and insufficient ADAR1p110 levels that could not induce editing activity. In the ADAR1-knockdown cell lines, EV-D68 was continuously detected for 15 passages. Sequencing showed a partial G-to-A transition at position 352 of 5′-UTR, which may be a consequence of a natural mutation accumulated by the virus during successive passages. Although the deaminase domain of ADAR1 is necessary for EV-D68 replication, whether ADAR1-mediated, direct A-to-I editing was performed needs to be further verified.

Although the primary function of dsRBDs is to mediate dimerization of ADAR1 to bind double-stranded RNA, this region is also involved in intracellular signaling pathways [38]. Our study demonstrated that ADAR1p110 could inhibit the activation of PKR signaling after EV-D68 infection. The reduction of endogenous ADAR1 resulted in increased phosphorylation of PKR and eIF2α, which in turn inhibited viral translation. In the absence of dsRBDs, ADAR1p110 no longer inhibited PKR phosphorylation, indicating that ADAR1 can regulate the PKR signaling pathway through its dsRBDs to promote EV-D68 replication. This mechanism was first reported in *Enterovirus*. Similarly, Pfaller et al. reported that ADAR1p150, but not ADAR1p110, inhibited PKR recognition of dsRNA, thereby promoting MeV replication [39]. ADAR1p150 has also been reported to interact with PKR in HIV-1-infected lymphocytes to promote viral replication [26, 27]. In this regard, we can say that both isoforms of ADAR1 can suppress the PKR pathway and contribute to viral replication.

**Fig. 5:**
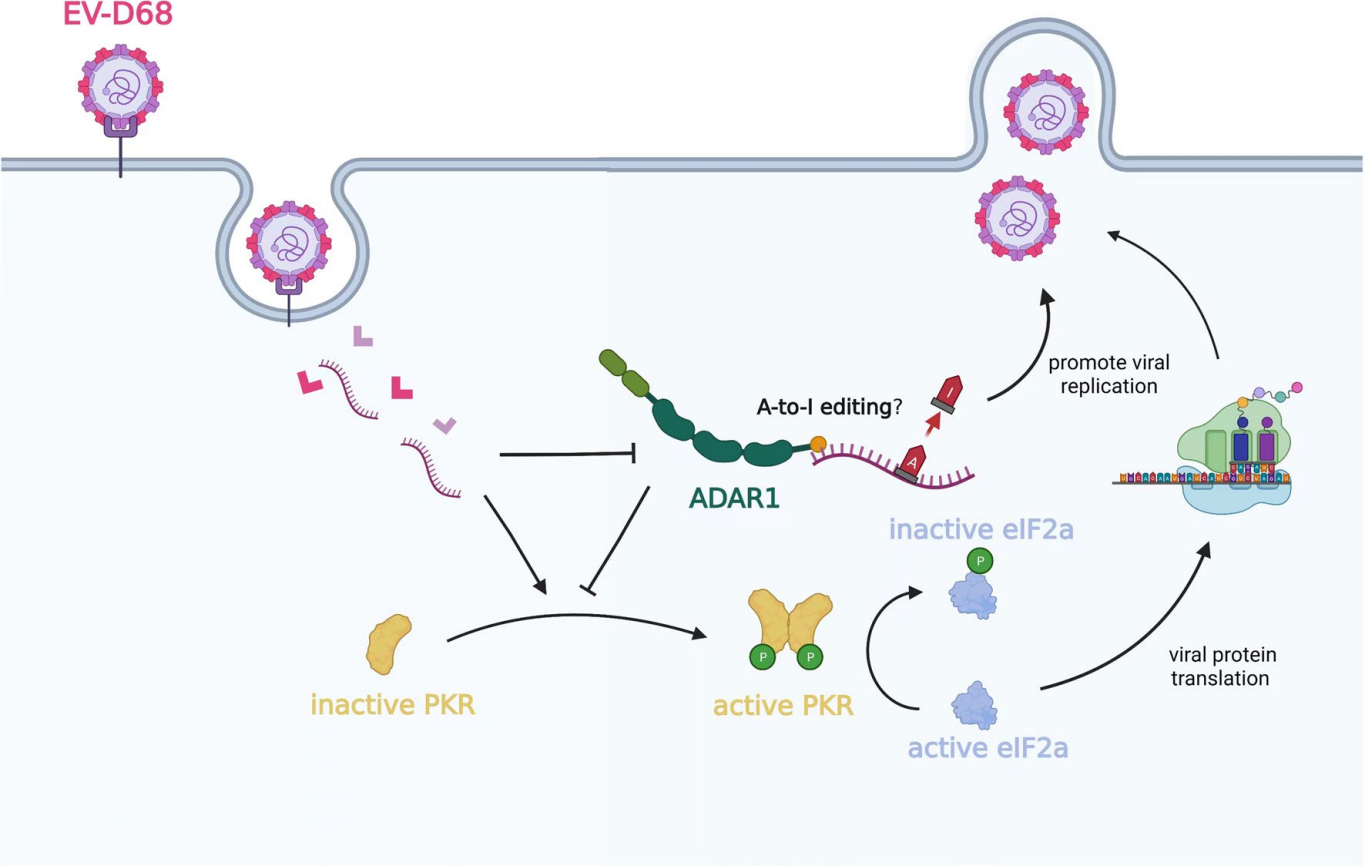
Schematic diagram of ADAR1 anti-EV-D68 mechanism. The promotion of EV-D68 by ADAR1p110 depends on its deaminase domain. In addition, ADAR1p110 also inhibits the phosphorylation of PRK through its dsRBD, thereby inhibiting the phosphorylation of eIF2a and promoting the translation of EV-D68 viral proteins.

Overall, we have demonstrated that ADAR1p110, but not ADAR1p150, promotes EV-D68 replication, and the ADAR1p110 pro-viral ability depends on both its deaminase domain and dsRBDs (Fig. 5). This study contributes to the understanding of the host innate immune mechanism against EV-D68 infection, and ADAR1 may serve as a potential target for anti-EV-D68 research.

## Conclusions

Taken together, our findings unravel the role of ADAR1 in EV-D68 infection and indicate that its deaminase domain is closely related to its pro-viral effect. In addition, the dsRBD region can inhibit the phosphorylation of PKR, thereby inhibiting the phosphorylation of eIF2α, and relieve the inhibition of viral protein translation. This is one of few reports on the role of ADAR1 in *Enterovirus* infection and the underlying mechanism and provides the latest example for further revealing the role of ADAR1 in different viral infections.

## Data Availability

All data generated during the current study are available upon request by contacting the corresponding authors.

## Abbreviations

EV-D68: *Enterovirus D68*
HPIV3: Human parainfluenza virus type 3
5’-UTR: 5’-Untranslated region
dsRBDs: Double-stranded RNA binding domains
PKR: Double-stranded RNA dependent protein kinase
p-PKR: Phosphorylated PKR
eIF2α: Eukaryotic Initiation Factor 2α
p-eIF2α: Phosphorylated eIF2α
Rd cells: Rhabdomyosarcoma RD cells
293 T cells: Human embryonic kidney 293 cells
ADAR1: Adenosine deaminase acting on RNA1
RT-qPCR: Quantitative reverse transcription PCR
TCID_50_: Median tissue culture infectious dose
IRES: Internal ribosome entry site
PBS: Phosphate buffered saline
HN protein: Hemagglutinin-neuraminidase protein
IFN: Interferon
ORF: Open Reading Frame
AFM: Acute flaccid myelitis
circRNAs: Circular RNAs
ADAR1 KD cell: ADAR1 stable knockdown cell
DMEM: Dulbecco’s modified Eagle’s medium
MOI: Multiplicity of infection
BSA: Bovine serum albumin
SD: Standard deviation
LLC-MK2 cell: Lilly Laboratories Cell-Monkey Kidney 2 cell
